# Structural and Rheological Characterization of a Sustainable Biopolymer: Arabinogalactan-Rich Mucilage from *Cereus hildmannianus*

**DOI:** 10.1021/acsomega.5c10974

**Published:** 2026-05-13

**Authors:** Aline Savam, Mariana C. de Oliveira, Marcos L. Bruschi, Rodrigo V. Serrato, Arildo J. B. de Oliveira, Regina A. C. Gonçalves

**Affiliations:** 1 Departamento de Farmácia, Programa de Pós-Graduação em Ciências Farmacêuticas, Laboratório de Biotecnologia de Produtos Naturais e Sintéticos, 42487 Universidade Estadual de Maringá, Maringá 87020-900, PR, Brazil; 2 Departamento de Farmácia, Programa de Pós-Graduação em Ciências Farmacêuticas, Universidade Estadual de Maringá, Maringá 87020-900, PR, Brazil; 3 Departamento de Bioquímica e Biologia Molecular, 28122 Universidade Federal do Paraná, Curitiba 80060-000, PR, Brazil

## Abstract

Natural polymers are valued for their low toxicity, biocompatibility, and biodegradability. Plant mucilage, a sustainable and cost-effective source rich in sugars and bioactive compounds, forms gel-like structures in water, enabling its applications as a viscosity enhancer, stabilizer, emulsifier, and biodegradable packaging alternative. Cacti, notably *Cereus hildmannianus* K. Schum., are resilient crops with traditional uses in medicine, cosmetics, nutraceuticals, and wastewater treatment. In this study, we extracted mucilage from *C. hildmannianus* cladodes, obtaining a 24% yield and separated it into a soluble fraction (SF, 22%) and an insoluble fraction (IF, 78%), both slightly acidic (4.75 and 5.44, respectively) and gel-forming. SF contained 32% total sugars, 9% reducing sugars, and 33% protein, whereas IF had a higher polysaccharide purity (44% total sugars, 4% reducing sugars, 7% protein). Chemical characterization (GC-MS, GC-FID, ^1^H/^13^C NMR, FTIR-ATR) identified the polysaccharide as a type I arabinogalactan with a galactose backbone and arabinose/rhamnose side chains. Rheological analysis revealed pseudoplastic behavior with concentration-dependent viscosity, comparable to guar and xanthan gums, highlighting its potential for food and pharmaceutical applications.

## Graphical abstract

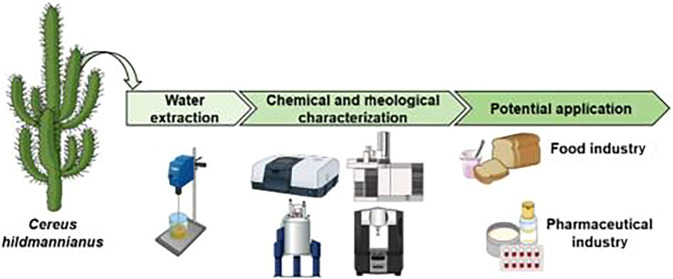

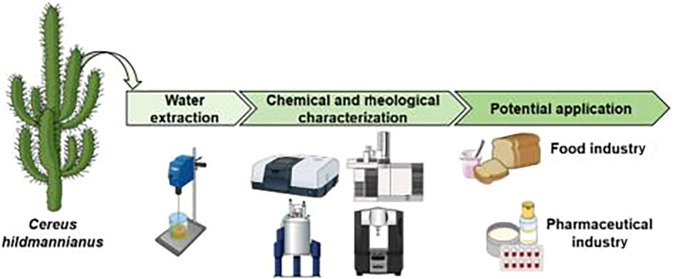

## 1 Introduction

Polymers are macromolecules that are formed by monomers joined by covalent bonds. They can be classified as natural or synthetic, depending on their origin. The composition of natural polymers may vary depending on the species, extraction method, seasonality, and drying methods used, and their applications are directly affected by this. These biopolymers have several applications in the pharmaceutical industry and have distinct functions in formulations, such as suspending, emulsifying, binding, and thickening agents.^1,2^ Also, natural polymers have been used in the food industry to improve the textural attributes as natural additives, fat replacers, and emulsifiers. They can even be incorporated into food packaging materials to enhance mechanical, thermal, and gas barrier properties.^3^

Currently, these compounds are gaining prominence due to their various advantages over synthetic polymers, such as low toxicity, biocompatibility, biodegradability, accessibility, and low cost. In this context, mucilage derived from *Mimosa pudica*, flaxseeds, and quince seeds, as well as natural gums such as xanthan gum, have been widely investigated and applied in the food, pharmaceutical, and biomedical fields.^4^ Furthermore, this material meets the profile of consumers who are currently looking for products that have a positive impact on their health, are ecologically correct, and are preferably plant-based.^5^

Among natural polymers, plant mucilage represents an accessible, sustainable, and cost-effective source. Furthermore, it constitutes a renewable raw material source, consistent with the seventh principle of green chemistry, which emphasizes the prioritization of biomass as a feedstock in the development of new technologies and industrial processes.^6,7^

Mucilages are complex, high molecular weight polysaccharides, which form viscous materials in water and are composed of sugar units, usually l-Ara, d-Gal, l-Ram, d-Xyl, and uronic acid. Proteins, lipids, bioactive compounds, and minerals are also observed in lower concentrations.^5^ Moreover, mucilages are essential components of plant cells that perform several functions, such as water and energy storage, and are found in various parts of the plant, including stems, fruits, leaves, and seeds.^8,9^

*Cactaceae* is one of the main sources of mucilage. The family comprises more than 1500 plant species divided into 127 genera. In Brazil, they are of significant importance, especially in the Northeast of the country, as they can survive the region’s semiarid climate.^10^

Climate change has intensified the need for resilient crops, such as cacti. Due to their low water requirements, adaptability to poor soils, and minimal agricultural inputs, cacti represent a sustainable cultivation option. Consequently, the use of cactus-derived products, such as mucilage, as alternatives to petroleum-based materials, contributes to environmentally responsible and economically viable solutions.^11^

*Cereus hildmannianus* K. Schum., *Cactaceae*, originally from South America, is popularly known as *Cacto*, *Mandacaru-de três-quinas*, or *Tuna*. This species has shown invasive occurrence associated with anthropogenic activities in regions such as France, Spain, Andorra, South Africa, Kenya, India, Vietnam, Taiwan, Australia, Portugal, Italy, and Sweden, where it typically survives in fine-textured soils, shaded environments, and moderate temperatures. In contrast, native populations occur in seasonal deciduous and semideciduous Atlantic and tropical forests, as well as in rocky outcrops, the Cerrado (Brazilian Savanna), and the Pampas biome.^12^ Therefore, understanding the chemical composition of individuals from specific geographic regions is essential, as environmental conditions can significantly influence the biochemical composition of the species.

This species is used in popular medicine for weight loss, cholesterol reduction, as a diuretic, against lung diseases, as an antirheumatic, cardiotonic, and as a healing agent.^12^ *In vitro* studies have revealed the gastroprotective effect of polysaccharides extracted from *C. hildmannianus* cladodes,^13^ along with the antioxidant activity^14^ and antifungal properties of its cladode extract.^15^

*C. hildmannianus* is a promising species due to its diverse bioactive compounds with potential applications in the pharmaceutical and food sectors. To the best of our knowledge, this study is the first to report the extraction and chemical and rheological characterization of its mucilage. These aspects are fundamental for evaluating its technological and functional potential since both its chemical composition and rheological behavior directly influence its applicability. Therefore, this study aimed to extract mucilage from *C. hildmannianus* cladodes and characterize it using colorimetric, chromatographic (GC–MS), and spectroscopic (FTIR-ATR, ^1^H, and ^13^C NMR) techniques, as well as steady shear rheological analysis.

## 2 Results and Discussion

### 2.1 Moisture Content of *C. hildmannianus* Cladodes

The cladodes of *C. hildmannianus* have a high moisture content (95.65%), which is common in the family, as water storage is regarded as an anatomical and physiological adaptation to survive drought.^16^ Similar results were observed for other *Cactaceae*, such as *Opuntia ficus-indica* (83–93%).^17,18^

### 2.2 Extraction Yield

The extraction yield was 23.96 ± 5.65%, calculated based on the dried cladode weight. The extraction method resulted in two distinct fractions: a white, flexible solid referred to as the insoluble fraction (IF), which appeared as a fine, homogeneous, and nonhygroscopic powder after freeze-drying, and a viscous liquid, the soluble fraction (SF), which yielded a fine, greenish, and highly hygroscopic powder after freeze-drying. The green coloration is likely associated with residual chlorophyll from the cladode, whereas the pronounced hygroscopicity may be attributed to its higher protein content, which can enhance the water-retention capacity of the mucilage structure.^9^ IF accounted for approximately 22% of the total extract mass, whereas SF represented 78%.

This extraction yield was similar to the values reported in the literature for other *Cactaceae*, such as those described by Petera et al.^19^ for *Cereus triangularis* (24%), Mannai et al.^17^ for *Opuntia ficus-indica* (19%), and Messina et al.,^18^ also for *O. ficus-indica* (12–26%), despite the application of more complex and costly techniques, such as hot extraction processes and microwave-assisted extraction.

The classical mucilage extraction method^10^ employed in this study proved to be environmentally friendly, in accordance with green chemistry principles,^6,7^ as it not only used low-toxicity solvents (water and ethanol) but also included rapid and direct steps, and did not require complex equipment or energy and time-consuming processes. Therefore, it is economically viable and easily applicable, facilitating scale-up and highlighting its potential for industrial applications, particularly in the pharmaceutical, food, and cosmetic sectors, where these kinds of materials are typically used as gelling agents and texture enhancers.^1–3^

To the best of our knowledge, this is the only study to report the formation or at least the valorization of two distinct fractions from the same extraction process. However, rather than indicating the presence of two chemically distinct polymers, the observed separation could be more appropriately interpreted as the solvent-induced reorganization of a single biopolymer system into two phases with different hydration states and compositions.

It is well established that the extraction yield is influenced not only by the extraction method but also by factors such as plant species, seasonality, and cladode age. In this context, simpler, safer, and more efficient extraction approaches are considered more attractive from a commercial standpoint.^20^

### 2.3 Chemical Characterization of Mucilage from *C. hildmannianus*

#### 2.3.1 pH and Solubility of Mucilage from *C. hildmannianus*

Both IF and SF exhibited slightly acidic pH values (5.44 and 4.75, respectively), consistent with the reported characteristics of *Cactaceae* mucilage, whose pH values typically range from 4.0 to 7.0. This parameter is particularly relevant for predicting mucilage behavior, as acidic conditions are generally associated with reduced solution viscosity,^10^ which may also account for the difference in viscosity between the two fractions. In addition, *Cactaceae* mucilages are commonly reported to exhibit good stability against pH, temperature, and salt concentrations, reinforcing their technological potential as robust biopolymers.^11^

Both fractions were fully soluble in water. In a 1% (w/v) aqueous solution, IF formed a whitish, highly viscous gel-like structure, whereas SF produced a greenish, translucent, and less viscous gel-like structure. The two fractions showed reduced solubility in NaOH and NaCl and were almost insoluble in citric acid and HCl. This behavior is consistent with that reported by Kalegowda et al.^8^ for *O. dillenii* mucilage and by Manhivi et al.^21^ for *Opuntia* spp. mucilage, which is expected, given the hydrophilic nature of this biopolymer.

#### 2.3.2 Colorimetric Analyses of Mucilage from *C. hildmannianus*

Colorimetric assays, followed by statistical comparison between IF and SF, revealed significant differences in total sugar, reducing sugar, and protein content. IF presented a total sugar content of 44.5 ± 1.71%, along with 4.1 ± 0.03% reducing sugars and 7.2 ± 0.23% protein. In contrast, SF exhibited a lower total sugar content (32.6 ± 0.34%), higher reducing sugar levels (9.0 ± 0.23%), and markedly higher protein content (33.2 ± 0.24%).

The sugar content observed in this study was similar to that reported by Andrade Vieira^22^ for *C. hildmannianus* (40%) mucilage, but lower than the values reported by Petera et al.^19^ for *C. triangularis* (85%) and Dick et al.^23^ for *O. monocantha* (80%). However, the sugar content in *Cactaceae* mucilage is known to vary widely, typically ranging from 40 to 97%, depending on factors such as plant age, irrigation conditions, extraction methods, and sample purity.^10^

The protein content, especially in SF, was significantly higher than the values observed in the literature for mucilage from other *Cactaceae*, such as *C. triangularis* (3%)^19^ and *Opuntia* spp. (3%),^21^ and even *Pereskia aculeata* (19%),^24^ which is known for its high protein content. Mensah et al.^25^ reported a higher protein content in nonpurified chia seed mucilage; therefore, in addition to species-related differences, the protein content observed in the present study may also be associated with sample purification.

Mucilages with higher protein content generally form more viscous hydrocolloids, as proteins enhance water retention. However, as seen in IF, the structuring capacity of the mucilage in *C. hildmannianus* appears to be more related to the polysaccharide. Higher protein content can also improve emulsifying capacity and reduce the surface tension of foams, thereby increasing the stability. These properties are particularly attractive for pharmaceutical and food applications, where such mucilages may be used as gelling agents, film-forming materials, and emulsion and foam stabilizers.^9,26,27^

#### 2.3.3 Monosaccharide Composition of Mucilage from *C. hildmannianus*

IF was mainly composed of galactose (Gal, 60.4%) and arabinose (Ara, 35.6%), with smaller amounts of rhamnose (Rha, 4.1%). Similarly, SF predominantly consisted of Gal (46.9%) and Ara (22.3%) but also contained a substantial proportion of glucose (Glc, 30.9%). Based on this monosaccharide profile, it is possible to infer that the polysaccharide can be classified as an arabinogalactan, a class of polysaccharides commonly found in plants, primarily composed of galactose and arabinose residues, and frequently with peptide chains attached, forming arabinogalactan proteins (AGPs).^28^

These polysaccharides are well-known for their emulsifying ability, foaming capacity, and gel-forming properties. Arabinose residues contribute predominantly to the hydrophilic character, water retention, and gelling behavior of mucilages.^28,29^

Galactose residues are mainly responsible for structural integrity, viscosity, adhesiveness, and gelation. The hydrophilic nature of mucilage is particularly important, as it enables the formation of a three-dimensional polymer network capable of trapping a large amount of water, resulting in a gel-like structure. These distinctive properties of arabinogalactan-rich mucilages make them especially suitable for applications in the pharmaceutical, cosmetic, and food industries.^28,29^

This composition is similar to that described by Tanaka et al.,^13^ who identified the monosaccharide composition of the polysaccharide in *C. peruvianus* as an arabinogalactan with the main chain consisting of galactose, arabinose, and rhamnose residues. Furthermore, several authors have also observed the predominance of arabinogalactan in the mucilages of other cacti, such as *Opuntia* sp.,^20,21^ *O. monocantha*,^23^ and *C. triangularis*,^19^ although none have reported the formation of two fractions.

#### 2.3.4 Nuclear Magnetic Resonance

The IF spectrum presented signals in the anomeric region, characteristic of arabinogalactans, notably at 4.98 ppm (α-Ara), 4.54–5.10 ppm (β-gal), and 5.33 ppm (α-Rha). Also, the signal found at 2.01 ppm was attributed to the −C*H*_3_ of the acetyl groups^13^ (Figure 1A).

**Figure 1:**
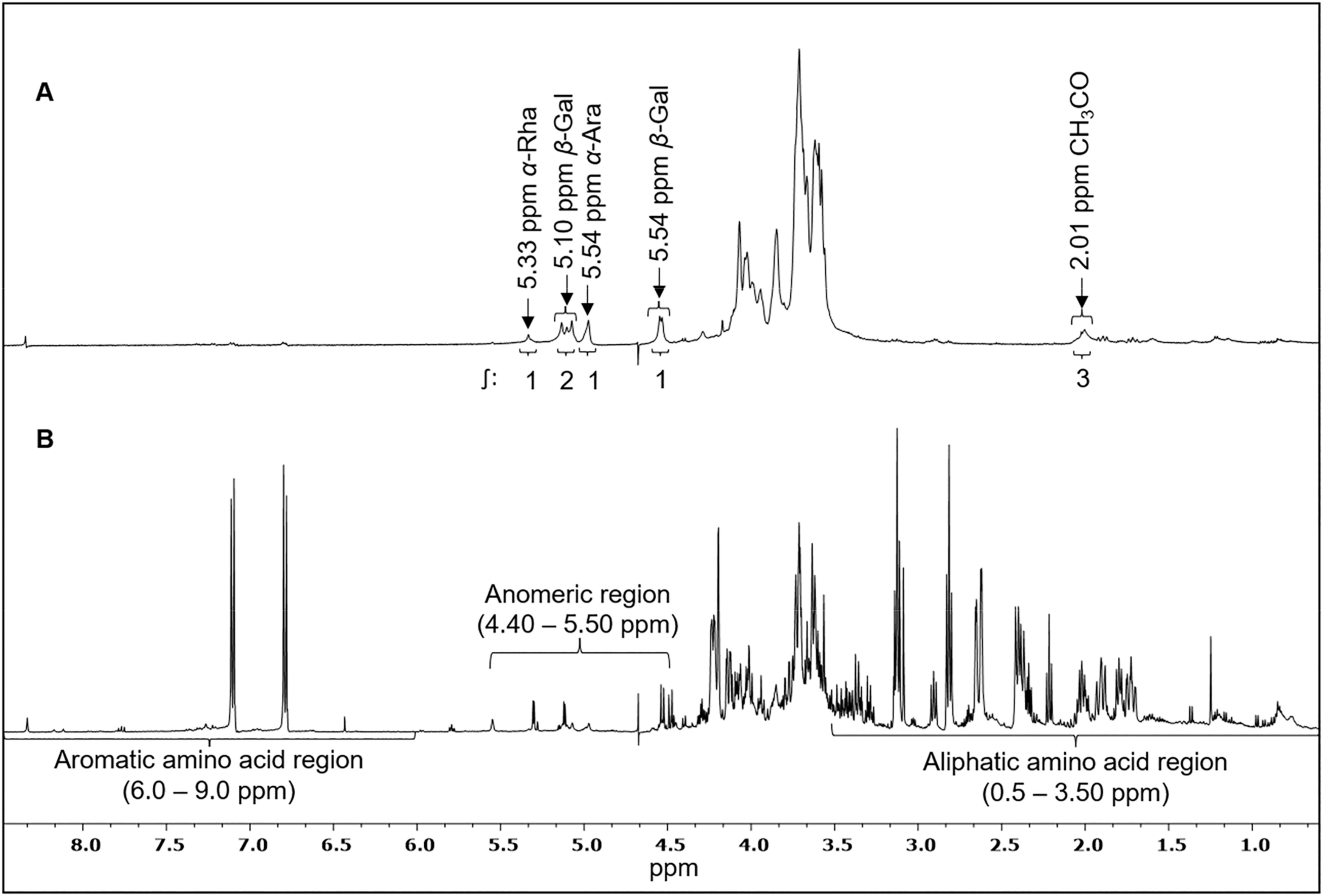
^1^H NMR spectra (500.13 MHz, D_2_O) of the insoluble (A) and soluble (B) fractions of mucilage from *C. hildmannianus* cladodes.

In the SF spectrum, intense signals in the aromatic amino acid region (6.0–9.0 ppm), abundant signals in the aliphatic amino acid region (0.5–3.5 ppm),^30^ and less intense signals in the anomeric hydrogen region (4.4–5.5 ppm) were observed,^13^ suggesting the predominance of proteins along with carbohydrates in the fraction (Figure 1B).

In ^13^C NMR spectrum of IF (Figure 2A), all the characteristic signals of an arabinogalactan were assigned as follows: β-Gal*p* typical peaks at 104.3 (C-1), 77.6 (C-4), 74.5 (C-5), 73.3 (C-3), 71.8 (C-2), and 60.7 (C-6) ppm; and α-Ara*f* peaks at 107.3 (C-1), 80.9 (C-2), 76.2 (C-3), 81.9 (C-4) e 60.4 (C-5) ppm.^13,31^ These suggest the presence of an arabinogalactan with a main β-Gal*p* backbone and branching α-Ara*f* units.

**Figure 2:**
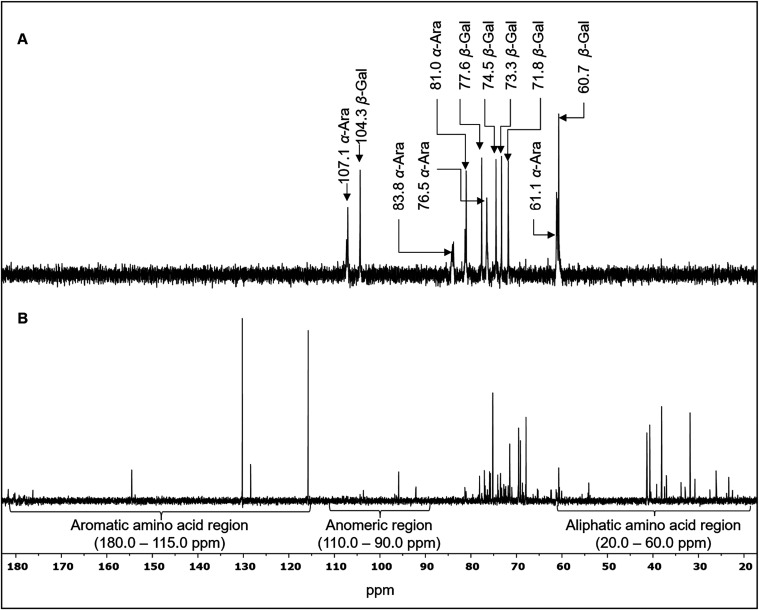
^13^C NMR spectra (125.7 MHz, D_2_O) of the insoluble (A) and soluble (B) fractions of mucilage from *C. hildmannianus* cladodes.

The spectrum acquired for SF (Figure 2B) demonstrated intense signals in the aliphatic (15.0–60.0 ppm) and aromatic (120.0–180.0 ppm) amino acid regions and exhibited low-intensity signals in the anomeric carbon region (90.0–110.0 ppm),^30^ and the typical amide carbonyl signals between 175 and 180 ppm,^32^ again suggesting the protein predominance in this fraction.

NMR analysis suggests that IF is a purer sample, predominantly composed of carbohydrates when compared to SF. The characteristic polysaccharide profile is consistent with other studies on *Cactaceae* mucilage, as described by Gheribi et al.^20^ in *O. ficus-indica*, Petera et al.^19^ in *C. triangularis*, and Kalegowda et al.^8^ in *O. dillenii*, though none have reported two distinct fractions, as observed in this study.

#### 2.3.5 Structure Analysis

As expected, *C. hildmannianus* mucilage exhibited a high estimated molecular weight (3.5 × 10^6^ Da), which falls within the typical range reported for cactus mucilage (2 × 10^5^ to 8 × 10^6^ Da).^10^ Linkage analysis was performed in IF, given that it was a purer fraction, mostly composed of polysaccharide chains, making it more suitable for this assay. The methylation profile was similar to that observed by Tanaka et al.^13^ in *C. peruvianus*, Campo-Grande et al.^33^ in *Bauhinia forficate*, and Hu et al.^34^ in polysaccharides from apples and tomatoes. The analysis of partially methylated alditol acetate (PMAA) derivatives obtained for the IF fraction suggests that the polysaccharide is most likely a type I arabinogalactan, characterized by a (1 → 4)-linked β-d-galactopyranosyl (β-Galp) main chain (Figure 3).

**Figure 3:**
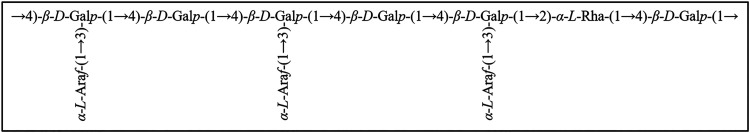
Proposed structure of type I arabinogalactan present in the mucilage of *C. hildmannianus*.

This structural assignment is supported by the predominance of 2,3,6-Me_3_-Gal*p* (27.1%), indicative of (1 → 4)-linked Gal*p* residues, with smaller contributions from 2,4-*O*-substituted rhamnopyranosyl (Rha*p*) residues (3-Me-Rha*p*, 1.3%), along with a substantial proportion of 2,6-Me_2_-Gal*p* (28.3%), revealing branching points at the *O*-3 position and suggesting a high branching degree, which may play a role in the structure–function relationship of the mucilage. Huang et al.^35^ reported that it is common to find arabinogalactans with a branching degree ranging from 38 to 100%, with one to four units per branch, typically composed of Ara or Gal, but Rha residues may also be present to a lesser extent.

The branching moieties are mainly composed of nonreducing terminal α-l-arabinofuranosyl (t-Ara*f*) units, as evidenced by the presence of 2,3,5-Me_3_-Ara*f* (21.0%) (Table 1). These methylation data corroborate the NMR spectroscopic results, which show a predominance of β-Gal*p* signals together with α-Ara*f* resonances, confirming a galactose-based backbone with arabinose side chains.

**Table 1:** Glycosyl Linkage Analysis of the *C. hildmannianus* Polysaccharides

retention time (min)	PMAA derivative	linkage	%
12.39	2,3,5-Me_3_-Ara*f*	t-Ara*f*	21.0
13.54	2,3,4-Me_3_-Ara*p*	t-Arap	0.6
14.65	2,3,4,6-Me_4_-Gal*p*	t-Galp	7.2
14.95	3-Me-Rha*p*	2,4-*O*-Rha*p*	1.3
15.44	2,3,6-Me_3_-Gal*p*	4-*O*-Gal*p*	27.1
15.70	2,4,6-Me_3_-Gal*p*	3-*O*-Gal*p*	5.1
16.30	2,6-Me_2_-Gal*p*	3,4-*O*-Gal*p*	28.3
17.46	2,4-Me_2_-Gal*p*	3,6-*O*-Gal*p*	9.4

#### 2.3.6 Attenuated Total Reflectance Fourier-Transform Infrared (FTIR-ATR) Spectroscopy

The SF spectrum (Figure 4) exhibited characteristic polysaccharide peaks. A broad band at 3209 cm^–1^ corresponds to hydroxyl groups, a C–H stretching peak at 2938 cm^–1^, a C–O stretch at 1045 cm^–1^, and a peak at 827 cm^–1^, characteristic of α-glycosidic bonds and associated with arabinogalactans, as described in the literature data.^29,36,37^ Carbonyl groups at 1583 cm^–1^ and 1377 cm^–1^, attributed to Amide I and Amide III of proteins, align with the values observed in the colorimetric and NMR analyses. The IF spectrum (Figure 3) had similar features with peaks at 3337, 2936, 1735, 1620, 1375, and 1040 cm^–1^, confirming its polysaccharide composition.^29,36,37^ These profiles are similar to mucilage from other *Cactaceae* species like *C. triangularis*,^19^ *O. dilleni*,^5^ and *O. ficus-indica*,^20^ which are also predominantly composed of arabinogalactans.

**Figure 4:**
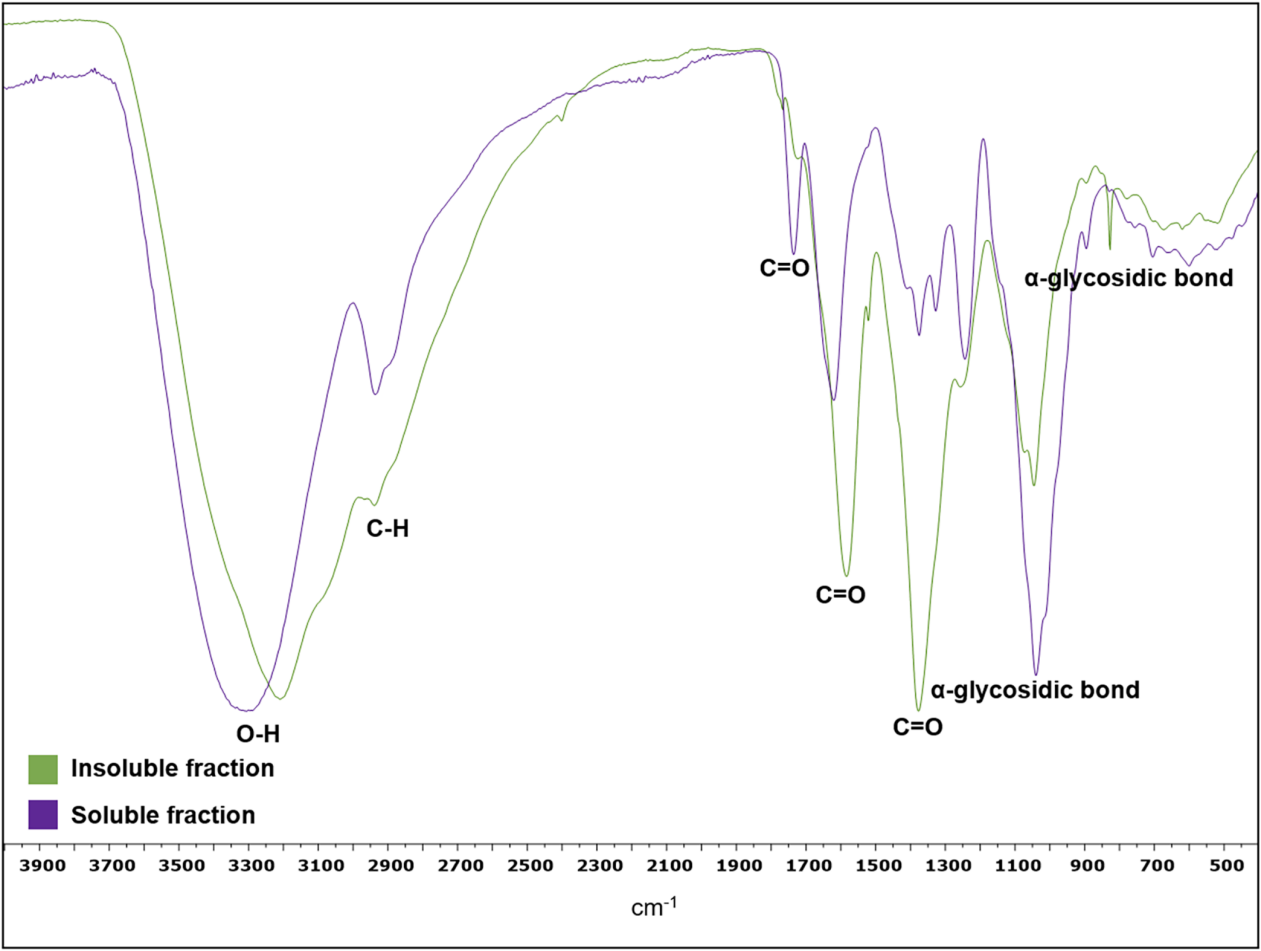
FTIR-ATR spectra of the insoluble and soluble fractions of mucilage from *C. hildmannianus* cladodes.

#### 2.3.7 Continuous Shear Rheology

Continuous shear rheological analysis was performed in order to investigate the flow behavior of the fluids. Understanding the flow rheological properties of polymers, such as mucilage, is essential for evaluating their applications and impacts on processing, final product quality, stability, and acceptability.

The Ostwald–de Waele rheological model adequately described the flow behavior of mucilage extracted from *C. hildmannianus* cladodes, with coefficients of determination higher than *R* ^2^ > 0.99. Both the IF and SF fractions exhibited Newtonian behavior (*n* = 1) at the lowest concentration evaluated (0.1%, w/v). However, from 0.25% (w/v) onward, both systems progressively exhibited pseudoplastic (shear-thinning) behavior (*n* < 1) (Table 2).

**Table 2:** Consistency Index (*K*) and Flow Behavior Index (*n*) from the Rheological Analysis of the Insoluble Fraction (IF) and Soluble Fraction (SF) of *C. hildmannianus* Mucilage Aqueous Solutions at 25 °C, at Different Concentrations^a^

	flow rheological parameters	
samples	*K* (Pa s)	*n*
SF 0.1%	0.001 ± 0.000	1.036 ± 0.031
SF 0.25%	0.005 ± 0.000	0.843 ± 0.016
SF 0.5%	0.024 ± 0.006	0.656 ± 0.040
SF 0.75%	0.046 ± 0.003	0.590 ± 0.008
SF 1.0%	0.067 ± 0.001	0.548 ± 0.019
IF 0.1%	0.003 ± 0.000	1.051 ± 0.049
IF 0.25%	0.025 ± 0.002	0.791 ± 0.011
IF 0.5%	0.146 ± 0.022	0.634 ± 0.026
IF 0.75%	0.192 ± 0.030	0.650 ± 0.024
IF 1.0%	0.264 ± 0.060	0.629 ± 0.029

a The analysis was performed in triplicate, and the results are expressed in mean ± standard deviation.

Pseudoplastic behavior is commonly observed in polymers and may be attributed to the alignment of their chains in the flow direction under increasing shear rates, which reduces intermolecular interactions and, consequently, decreases the apparent viscosity.^38^ This behavior is particularly important for pharmaceutical systems during administration, ensuring ease of application, uniform spreading across the entire region, and improved site retention once the original viscosity is restored.

SF displayed greater resistance to applied shear, maintaining structural integrity at shear rates up to 2000 s^–1^, whereas the IF fraction exhibited structural breakdown at approximately 500 s^–1^ for concentrations higher than 0.25% (Figure 5A,B). This result indicated that SF is better able to withstand high shear forces, a property that may be advantageous during raw material processing and for ensuring product stability during handling and application. This enhanced shear resistance may be associated with the higher protein content of the SF fraction, which contributes to increased matrix hydration^9^ and may reinforce the network, potentially reducing shear-induced disruption.

**Figure 5:**
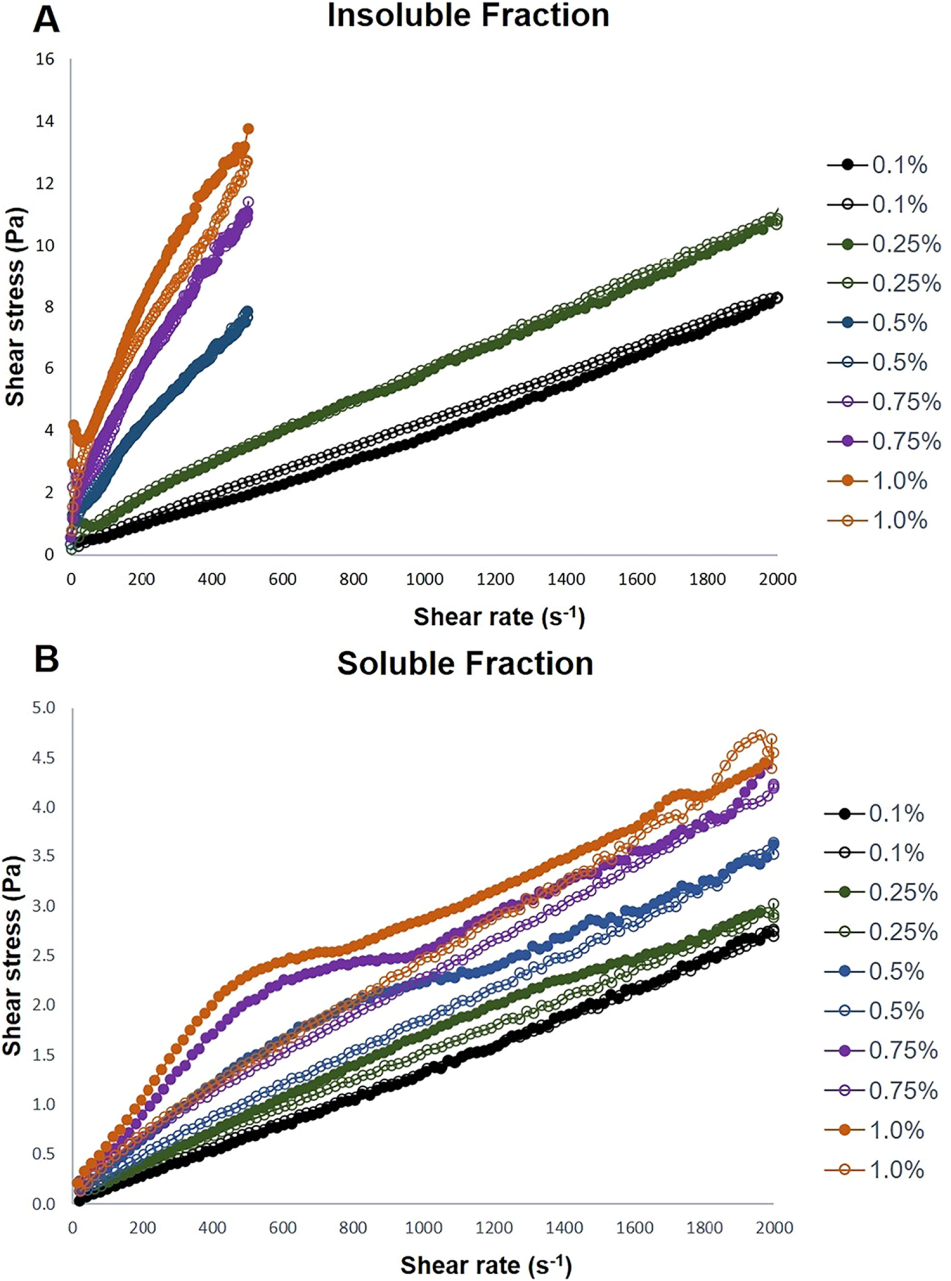
Flow curves of the insoluble (A) and soluble (B) fractions at different concentrations. The closed symbols represent the up curve, and the open symbols represent the down curve. Standard deviations have been omitted for clarity, despite the relative standard deviation of the replicate analysis being <10% in all cases.

Increasing the concentration from 0.1% to 1.0% (w/v) resulted in statistically significant differences in the consistency index values (*p* < 0.05) between the concentrations within both fractions, although the magnitude of this increase was less pronounced for SF. Moreover, IF consistently exhibited higher apparent viscosity than SF at concentrations of 1.0%, 0.75%, and 0.5% (w/v) (*p* < 0.05), while no significant differences were observed at the remaining concentrations.

From a functional perspective, these results suggest that IF may act as a more effective viscosity-enhancing agent, whereas SF, despite its lower viscosity, exhibits greater shear stability. Consequently, SF may be more suitable for applications involving high shear conditions, such as pharmaceutical systems that can undergo extrusion during the preparation process or administration. IF may be better suited for gel-forming applications like semisolid drug delivery systems (i.e., gels and emulgels). These properties highlight the remarkable versatility and potential applications of the obtained material.

With regard to time-dependent behavior, SF exhibited thixotropic behavior at all concentrations tested, whereas IF exhibited variable behavior, showing either thixotropic or rheopectic tendencies depending on the shear rate and concentration. The hysteresis areas observed for them indicate time-dependent recovery, which may be very useful during the administration of pharmaceutical systems and drug release. Thixotropy can enable a faster recovery of system viscosity after administration, and the hysteresis area can contribute to the rapid release of the drug. This variability indicates that the mucilage matrix is capable of structural reorganization after shear, reflecting the dynamic nature of the intermolecular interactions within the system.

This behavior is consistent with the reports on mucilage from *O. dillenii* ^8^ and *O. ficus-indica*,^39^ as well as with the rheological behavior of widely used commercial thickeners, such as xanthan gum, and other well-studied plant mucilages, including flaxseeds and chia seeds.^4^

#### 2.3.8 Scanning Electron Microscopy

Regarding their microstructure, both SF and IF exhibited particles with irregular size and shape distributions, where smaller particles were aggregated with larger ones. Nevertheless, the SF fraction appeared more amorphous, with a sponge-like characteristic (Figure 6A,B), while IF displayed a more crystalline and brittle appearance (Figure 6C,D). This difference reflects the fraction constitution and the reason why the polymer structure of IF withstands less shear stress.

**Figure 6:**
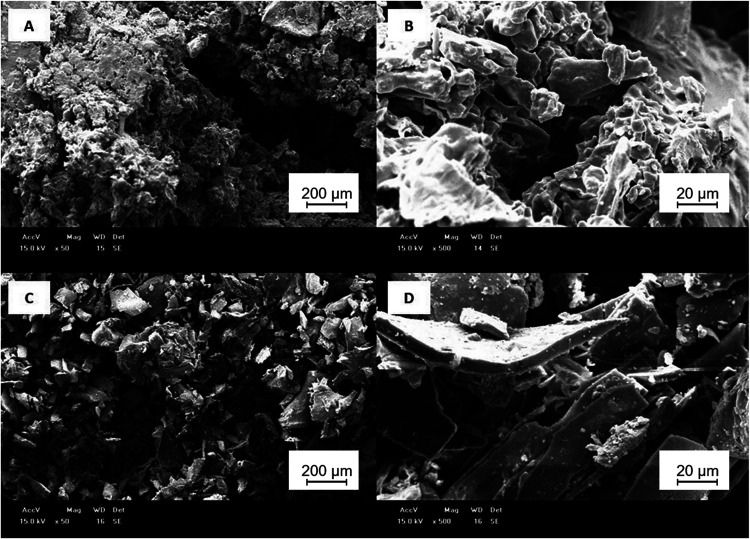
Scanning electron microscopy images of the soluble fraction at 50× (A) and 500× magnification (B) and the insoluble fraction at 50× (C) and 500× magnification (D) of mucilage from *C. hildmannianus* cladodes.

The microstructure of the mucilage powder also depends on the extraction conditions and purification and drying methods used. These characteristics were similar to those observed by Dick et al. and Mannai et al. when studying the mucilage of *O. monocantha* and *O. ficus-indica*, respectively.

## 3 Conclusion

The extraction methodology demonstrated economic viability, operational simplicity, and environmental compatibility, reinforcing its potential for industrial-scale implementation. Chemical characterization confirmed that the mucilage primarily comprised arabinogalactan-type polysaccharides and proteins, with distinct compositional and rheological differences between the solvent-induced fractions. This work, for the first time, reveals a chemically distinct, fractionated mucilage from *C. hildmannianus*, in which the soluble fraction provides structural stability, while the insoluble fraction offers partially purified polysaccharides with higher viscosity, enabling tailored applications in the food and pharmaceutical sectors. In summary, these results demonstrate that *C. hildmannianus* mucilage is a promising sustainable biopolymer; however, further investigations focused on its technofunctional performance, protein composition, and structure are necessary for its industrial application.

## 4 Materials and Methods

### 4.1 Materials

The following materials were used in this study. Ethanol 96° (CAS 64-17-5, 92.8%, Itajá), NaCl (CAS 7647-14-5, 99.5%, Fmaia), NaOH (CAS 1310-73-2, 99%, Biotec), citric acid (CAS 77-92-9, 99.5%, Qhemis), phenol (CAS 108-95-2, Êxodo Científica), sulfuric acid (CAS 7664-93-9, 97.3%, J. T. Baker), dinitrosalicylic acid (CAS 609-99-4, 98%, Inlab), NaCO_3_ (CAS 497-19-8, 99.5%, Anidrol), CuSO_4_ (CAS 7758-99-8, 98%, LabSynth), Folin-Ciocalteu reagent (CAS 10213-10-2, 10102-40-6, Dinâmica), deuterium oxide (CAS 7789-20-0, 99.9%, Sigma-Aldrich), sodium and potassium tartrate (CAS 6381-59-5, Ecibra), pyridine (CAS 110-86-1, 100%, LabSynth), acetic anhydride (CAS 108-24-7, 100%, LabSynth), dimethyl sulfoxide (CAS 67-68-5, 99.7%, Merck), trifluoroacetic acid (CAS 76-05-1, 99%, Sigma-Aldrich), iodomethane (CAS 74-88-4, 99%, Sigma-Aldrich), sodium borohydride (CAS 16940-66-2, Neon), sodium borodeuteride (CAS 15681-89-7, Sigma-Aldrich), glucose (≥99%, Sigma-Aldrich), bovine albumin (≥99%, Sigma-Aldrich), and dextrans (Sigma-aldrich, purity ≥99%).

### 4.2 Plant Material

Cladodes of *C. hildmannianus* were collected in October 2022 on the campus of the State University of Maringá (UEM), Maringá, Paraná, Brazil (Latitude 23° 40′ 52″ S, Longitude 51° 94′ 19′′ W, Altitude of 517 m). The species, identified by Dr. Daniela Cristina Zappi from the Vale Technological Institute (ITV), is registered in SisGen (A05B398), and a specimen (HUEM 36127) was deposited in the UEM Herbarium. The spines of the collected cladodes were removed, and they were cleaned with neutral detergent and abundant potable and purified water. Afterward, the cladodes were diced into smaller pieces and frozen.

### 4.3 Moisture Content of *C. hildmannianus* Cladodes

The moisture content of the cladodes was determined according to the seventh Edition of the Brazilian Pharmacopeia method.^40^ Approximately 5 g of the cladodes was cut into 3 mm fractions and dried in an oven at 105 °C for 5 h until a constant weight. The moisture content was calculated using eq 1:

1 $$m\mathrm{oisture content}\;\left(\%\right)=\left(\frac{\mathrm{DW}}{\mathrm{FW}}\right)\times 100$$

where DW stands for Dry Weight, and FW stands for Fresh Weight.

### 4.4 Mucilage Extraction from *C. hildmanniannus* Cladodes

The frozen cladode pieces (100 g per batch) were soaked in purified water (1:5, w/v, pH 5.66, room temperature) and stirred for 1 h on a mechanical stirrer (713D, Fisatom, Brazil) for the release of the mucilage, which was then separated from the residue by using a strainer. The extract was precipitated with three volumes of 96° ethanol at 4 °C for 16 h. After precipitation, two fractions were formed (Scheme 1): a viscous liquid (soluble fraction (SF)) and a white and flexible solid material (insoluble fraction (IF)). They were freeze-dried, and the yield extraction was calculated considering the amount of dried powder of the combined fractions and the amount of utilized dried cladode weight.^7,8^

**Scheme 1:**
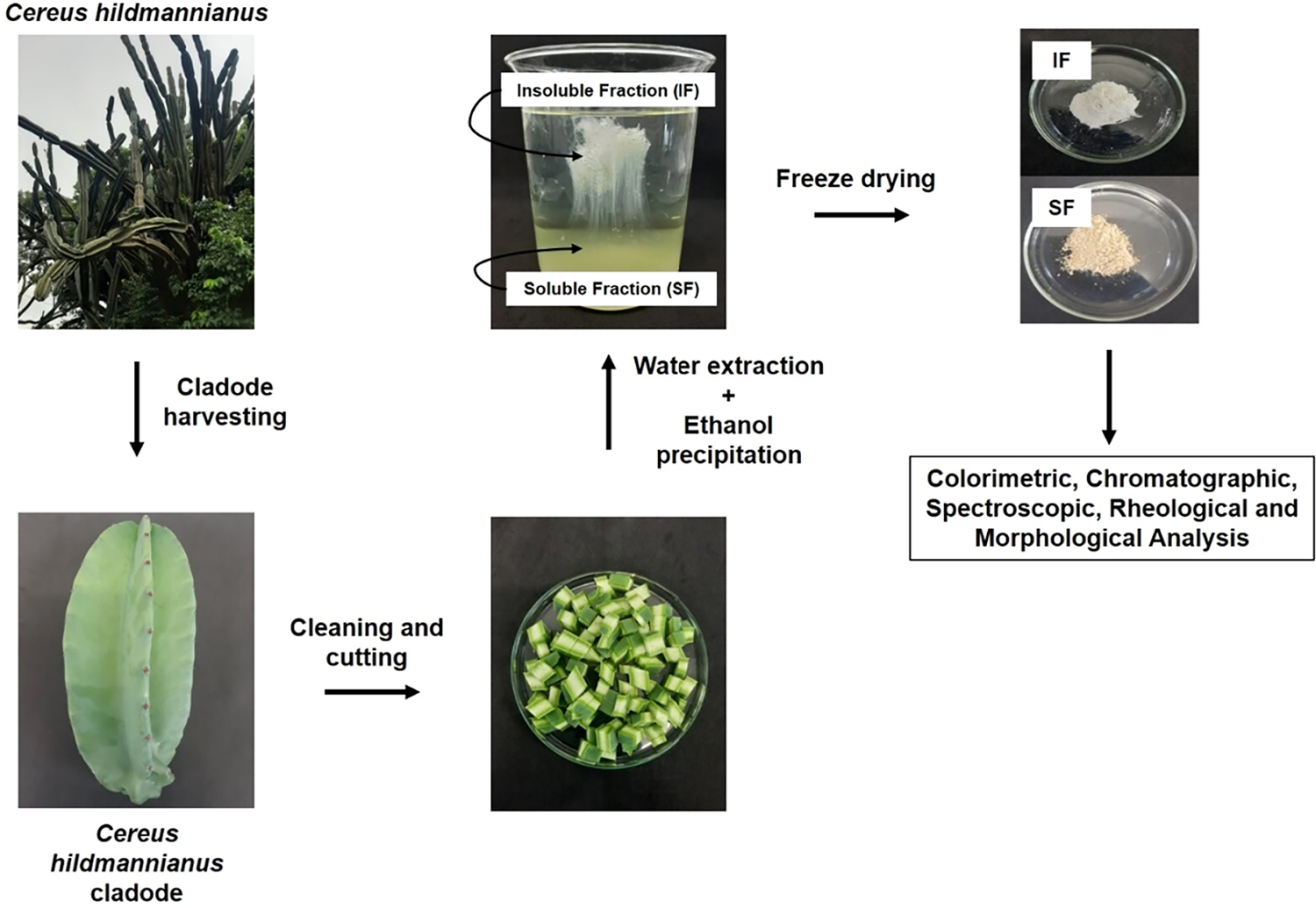
Schematic Representation of the General Methodology Used in This Study

### 4.5 pH and Solubility of Mucilage from *C. hildmannianus* Cladodes

The pH of the IF and SF was measured with a pH meter (PG1800, Gehaka, Brazil) in a 1% (w/v) solution. The solubility was evaluated in purified water, sodium chloride (1 M NaCl), sodium hydroxide (1 M NaOH), and citric acid (1 M C_6_H_8_O_7_) with 1% (w/v) solutions of IF and SF.^8^

### 4.6 Colorimetric Assay

All of the analyses were performed with IF and SF, in triplicate, using an Agilent Cary 60 UV–vis spectrophotometer.

Total sugars were measured according to Dubois et al.^41,42^ with modifications. To calculate the total sugars, an analytical curve (Figure S1) was constructed with a glucose standard, and the results were expressed as percentages.

The dosage of reducing sugars was determined according to Miller et al.^42,43^ with modifications. To calculate the reducing sugars, an analytical curve (Figure S2) was constructed using a glucose standard, and the results are expressed as percentages.

Protein content was determined according to Lowry et al.^44,45^ (Biuret method, modified), with modifications. To calculate the protein content, an analytical curve (Figure S3) was constructed with a bovine albumin standard and the results were expressed as percentages.

### 4.7 ^1^H and ^13^C Nuclear Magnetic Resonance Analyses

SF and IF were diluted in 600 μL of 99.9% D_2_O and maintained at 50 °C for 24 h to exchange hydrogen with deuterium (^1^H for ^2^H). The samples were then freeze-dried. The dry residues were solubilized in 600 μL of 99.9% D_2_O and subjected to NMR analyses, which were conducted at 298 K using a Bruker Advance HD III spectrometer operating at 500.13 MHz for the ^1^H nucleus and 125.7 MHz for the ^13^C nucleus, using the standard pulse sequences that are available in the Bruker software. Chemical shifts (δ) were expressed in parts per million (ppm), and the spectra were interpreted by comparison with the literature data.

### 4.8 Fourier-Transform Infrared Spectroscopy with Attenuated Total Reflection (FTIR-ATR) Analyses

Dried SF and IF were subjected to FTIR-ATR analysis, carried out on a Vertex 70v spectrometer (Bruker), in the spectral range of 4000–400 cm^–1^. The spectra were interpreted by comparison with literature data.

### 4.9 Monosaccharide Composition of the Mucilage from *C. hildmannianus*

To analyze the monosaccharide composition, 2 mg of SF and IF were hydrolyzed with 1 mL of 1 M TFA at 100 °C for 8 h. After evaporation, the material was solubilized in 0.5 mL of water, and NaBH_4_ was added in excess for reduction to occur over 12 h. Methanol was repeatedly added to the solution and evaporated (3 times) with airflow to remove boron as trimethyl borate before freeze-drying. Next, 1.0 mL of Ac_2_O-pyridine (1:1, v/v) was added to the samples, which were kept at room temperature for 12 h. The resulting alditol acetate derivatives were extracted by adding 1 mL of CHCl_3_, followed by repeated washing with a 5% copper sulfate solution. The chloroform extract containing the alditol acetate derivatives was recovered and evaporated for subsequent analysis by GC-FID.^46,47,22^ A DB-1 capillary column (30 m × 0.25 mm) (Agilent Technologies) was used, and the temperature program was adjusted from 100 to 140 °C (5 °C min^–1^), increasing to 240 °C and maintained for 10 min. Helium was used as the carrier gas (grade 5.0, 1.0 mL min^–1^). The injector was maintained at 260 °C, and the spectra were obtained using a FID (flame ionization detector) with a hydrogen-air flame (260 °C). The samples were identified and quantified based on the retention time of the monosaccharide standard.

### 4.10 Molecular Weight

The molecular weight (*M*_w_) of the polysaccharide (IF) was estimated by high-performance size-exclusion chromatography (HPSEC). The peak elution time was compared with a calibration curve (Figure S4) obtained with standard dextrans of 266, 124, 72.2, 40.2, 17.2, and 9.4 kDa. HPSEC analyses were performed using four Ultrahydrogel columns in series, with exclusion sizes of 7 × 10^6^, 4 × 10^5^, 8 × 10^4^, and 5 × 10^3^ Da, and a refractive index detector. The eluent was 0.1 M aq. NaNO_2_ containing 200 ppm aq. NaN_3_ at 0.6 mL/min. Samples and dextrans at a concentration of 1 mg/mL were filtered through a 0.22 μm membrane and injected (100 μL loop).

### 4.11 Methylation Analysis

For the linkage analysis of monosaccharide units, freeze-dried IF (5 mg) was fully methylated with 1 mL iodomethane (CH_3_I) after solubilization in 3 mL dimethyl sulfoxide (DMSO) with an excess of powdered NaOH.^48^ Permethylated polysaccharides were recovered after partitioning with CHCl_3_:H_2_O (1:1, v/v), and the organic phase was collected. The sample was then hydrolyzed with 1 mL of TFA (1 M, 8h, 100 °C), dried, and resolubilized in water (1 mL) for reduction with NaBD_4_ overnight. Sequential washings with MeOH were made, followed by evaporation until the sample was dried. Acetylation was then performed using pyridine:Ac_2_O (1:1 v/v) for 1 h at 120 °C to form permethylated alditol acetate monosaccharide derivatives (PMAA).^49^ PMAA were recovered in the organic phase after partitioning with CHCl_3_ (2 mL) and sequential washes with aqueous CuSO_4_ 5% solution. The PMAA was analyzed by GC-MS on a Shimadzu chromatographer (Nexus GC-2030) equipped with a DB-5 MS capillary column (30 m × 0.25 mm × 1.00 μm) (Agilent Technologies) coupled to a mass analyzer (QP2020 NX). The temperature program began at 100 °C for 3 min, then increased at a rate of 40 °C/min to 220 °C, 250 °C, and finally 280 °C, with a 3 min hold at each intermediate step. The GC peaks and MS fragments of PMAA (Figures S5 and S6, respectively) were identified according to previous studies.^22,50,51^

### 4.12 Continuous Shear Rheological Analyses

The flow analyses of diluted SF and IF at different concentrations (0.1, 0.25, 0.5, 0.75, and 1.0% w/w, diluted in purified water) were performed using a gradient and controlled shear stress MARS II (Thermo-Haake, Germany) in flow mode at a temperature of 25 ± 1 °C, with parallel cone–plate geometry of 60 mm in diameter and an angle of 1°, separated by a fixed distance of 0.052 mm. The samples were carefully applied to the plate, ensuring minimal shear, and a resting time of 60 s was allowed before starting each analysis. A shear gradient ranging from 0 s^–1^ to 2000 s^–1^ was applied to the samples over a period of 150 s. The upward flow curves and their parameters were analyzed using Waele’s Ostwald equation (Power Law) with RheoWin software (Thermo-Haake, Germany).

### 4.13 Scanning Electron Microscopy

Morphological characterization of SF and IF was carried out using a Scanning Electron Microscope (SEM) (Shimadzu), following standard procedures. The samples were adhered to sample holders (stubs) by using conductive carbon fiber tape suitable for electron microscopy. The samples were covered with a thin layer of gold in a Sputter Coater metalizer (Baltec) and subsequently observed under a scanning electron microscope to study the surface of the samples.

### 4.14 Statistical Analysis

All assays were conducted in triplicate, and the obtained data were analyzed by Analysis of Variance (ANOVA) followed by the Tukey post-hoc test, considering *p-*value < 0.05 to be significant.

## Supplementary Material



## Notes

Analytical curve for glucose standard solutions, obtained using the Dubois (phenol-sulfuric) method for total sugar determination; analytical curve for glucose standard solutions, obtained using the Miller (DNS) method for reducing sugar determination; analytical curve for albumin standard solutions, obtained using the Lowry method for total protein determination; analytical curve for dextran standard solutions, obtained using high-performance size-exclusion chromatography (HPSEC); chromatogram of partially methylated alditol acetates (PMAAs); EI mass spectra of PMAAs. (PDF)
