# Supplementary material for: Structural and Rheological Characterization of a Sustainable Biopolymer: Arabinogalactan-Rich Mucilage from Cereus hildmannianus
Source: ACS Omega. 2026 May 13;11(20):29449–60. doi: 10.1021/acsomega.5c10974 (PMC13216979; doi:10.1021/acsomega.5c10974)

Suporting Information

Structural and Rheological Characterization of a Sustainable Biopolymer: An Arabinogalactan-Rich Mucilage from *Cereus hildmannianus*

Aline Savam[a], Mariana C. de Oliveira[a], Marcos L. Bruschi[a], Rodrigo V. Serrato[b], Arildo J. B. de Oliveira[a], Regina A. C. Gonçalves*[a]

[a]Departamento de Farmácia, Universidade Estadual de Maringá, Maringá, PR, Brazil [b]Departamento de Bioquímica e Biologia Molecular, Universidade Federal do Paraná, Curitiba, PR, Brazil

*Corresponding author: Regina Aparecida Correia Gonçalves (racgoncalves@uem.br)

Figure S1. Analytical curve for glucose standard solutions in the range of 10–90 µg/mL, obtained using the Dubois (phenol-sulfuric) method for total sugar determination. A coefficient of determination (R^2) higher than 0.99 and a coefficient of variation lower than 5% were accepted. The linear regression equation is shown in the graph.

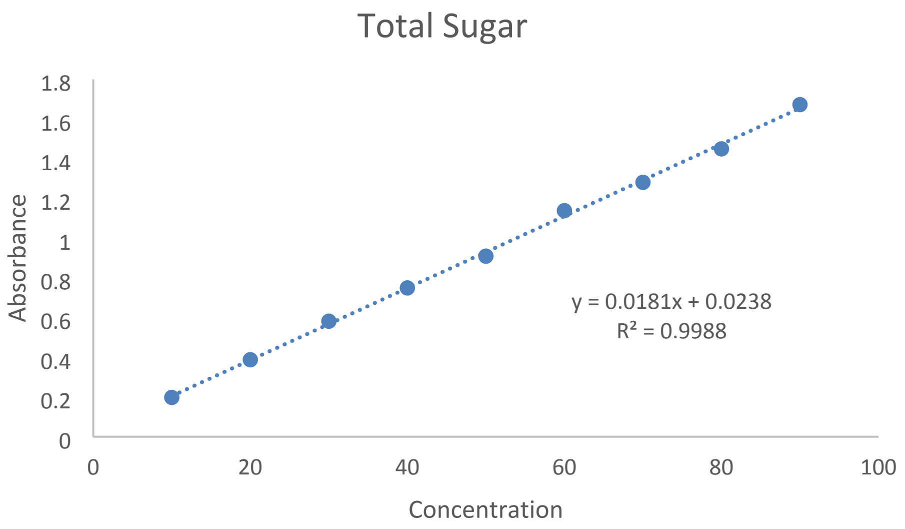

Figure S2. Analytical curve for glucose standard solutions in the range of 45–450 µg/mL, obtained using the Miller (DNS) method for reducing sugar determination. A coefficient of determination (R^2) higher than 0.99 and a coefficient of variation lower than 5% were accepted. The linear regression equation is shown in the graph.

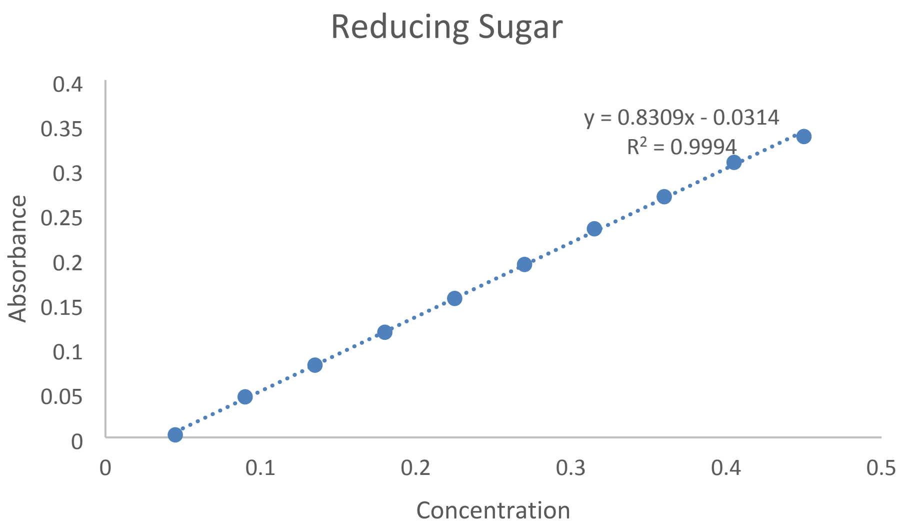

Figure S3. Analytical curve for albumin standard solutions in the range of 20–100 µg/mL, obtained using the Lowry method for total protein determination. A coefficient of determination (R^2) higher than 0.99 and a coefficient of variation lower than 5% were accepted. The linear regression equation is shown in the graph.

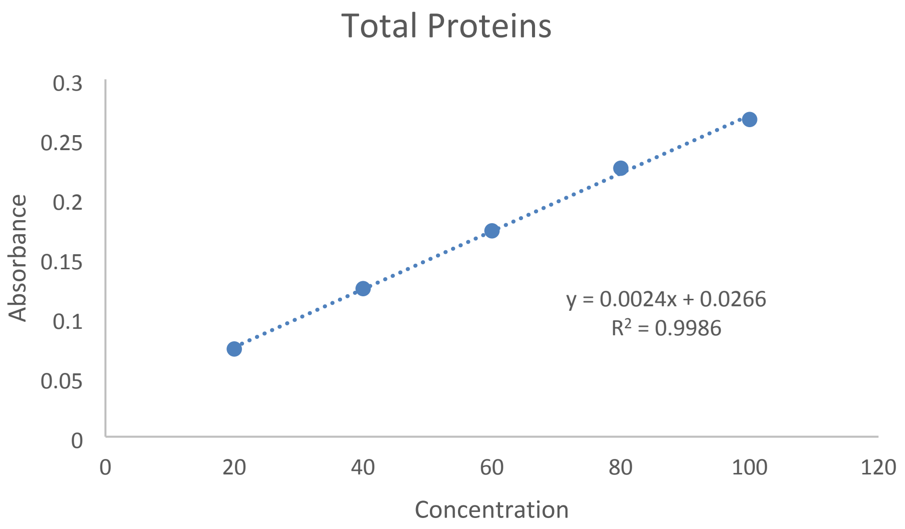

Figure S4. Analytical curve for dextran standard solutions in the range of 9–266 KDa, obtained by high performance size exclusion chromatography (HPSEC) for molecular weight estimation. A coefficient of determination (R^2) higher than 0.98 and a coefficient of variation lower than 5% were accepted. The linear regression equation is shown in the graph.

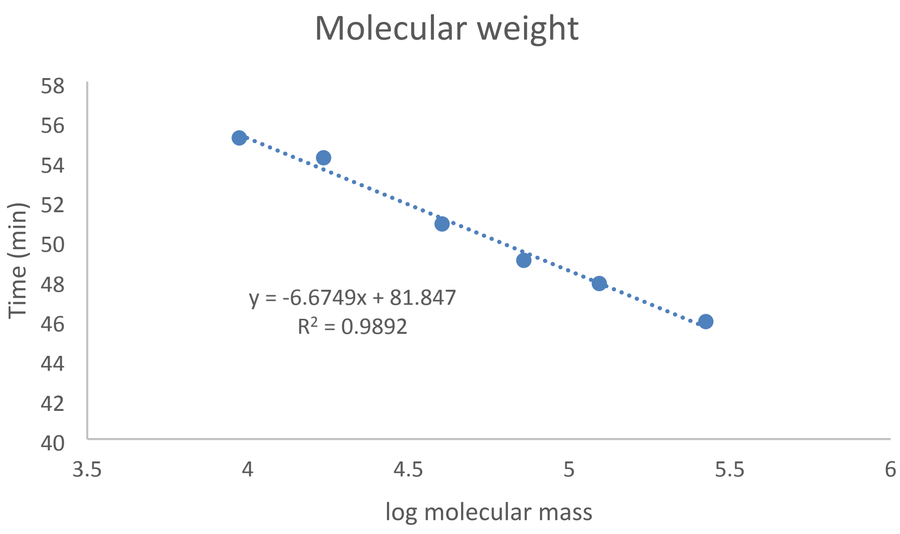

Figure S5. Chromatogram of partially methylated alditol acetates (PMAAs) obtained from the insoluble fraction of mucilage extracted from *Cereus hildmannianus* cladodes.

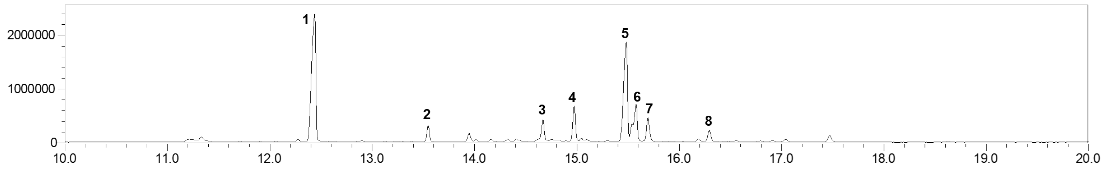

Figure S6. EI Mass spectra of partially methylated alditol acetates (PMAAs) of the insoluble fraction of mucilage from *Cereus hildmannianus* cladodes. The mass spectra are numbered according to their order of appearance in the chromatogram.

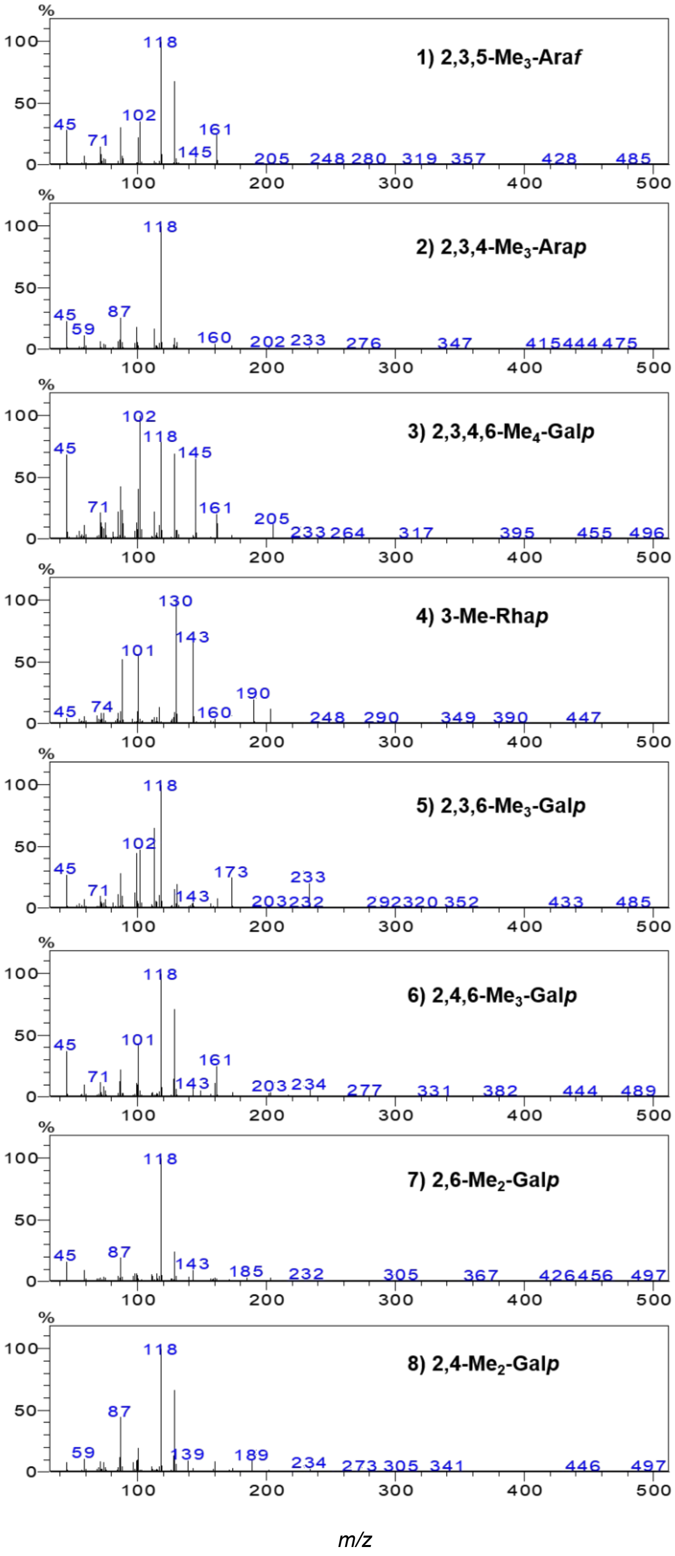

Supplement: Supplementary file 1 [file ao5c10974_si_001.pdf]
